# Using “outbreak science” to strengthen the use of models during epidemics

**DOI:** 10.1038/s41467-019-11067-2

**Published:** 2019-07-15

**Authors:** Caitlin Rivers, Jean-Paul Chretien, Steven Riley, Julie A. Pavlin, Alexandra Woodward, David Brett-Major, Irina Maljkovic Berry, Lindsay Morton, Richard G. Jarman, Matthew Biggerstaff, Michael A. Johansson, Nicholas G. Reich, Diane Meyer, Michael R. Snyder, Simon Pollett

**Affiliations:** 1 Johns Hopkins Center for Health Security, Baltimore, MD 21202 USA; 2 Department of Defense, Fort Detrick, MD 21702 USA; 3 MRC Centre for Global Infectious Disease Analysis, School of Public Health, Imperial College, London, UK; 4 National Academies of Sciences, Engineering, and Medicine, Washington, DC 20006 USA; 5 Cherokee Nation Strategic Programs, Tulsa, OK 74116 USA; 6 Global Emerging Infections Surveillance, Armed Forces Health Surveillance Branch, Silver Spring, MD 20904 USA; 7 Department of Preventive Medicine & Biostatistics, Uniformed Services University, Bethesda, MD 20814 USA; 8 Viral Diseases Branch, Walter Reed Army Institute of Research, Silver Spring, MD 20910 USA; 9 Department of Global Health, Milken Institute School of Public Health, George Washington University, Washington, DC 20037 USA; 10 Influenza Division, Centers for Disease Control & Prevention, Atlanta, GA 30333 USA; 11 Division of Vector-Borne Diseases, Centers for Disease Control & Prevention, Atlanta, PR USA; 12 Department of Biostatistics and Epidemiology, University of Massachusetts Amherst School of Public Health and Health Sciences, Amherst, MA 01003 USA; 13 Marie Bashir Institute for Infectious Diseases & Biosecurity, University of Sydney, Sydney, NSW Australia

**Keywords:** Computational models, Mathematics and computing, Infectious diseases

## Abstract

Infectious disease modeling has played a prominent role in recent outbreaks, yet integrating these analyses into public health decision-making has been challenging. We recommend establishing ‘outbreak science’ as an inter-disciplinary field to improve applied epidemic modeling.

## The enduring threat of epidemics and the promise of models

In 2018–2019, the Ebola virus disease emergency in the Democratic Republic of Congo continues to prompt fresh reflections on how to optimize epidemic response^1^. ‘Lessons learned’ from previous outbreaks often focus on the development and deployment of medical countermeasures, such as vaccines and antivirals. However, additional tools can aid in outbreak response. Mathematical and statistical modeling has repeatedly proven to be a valuable resource in targeting outbreak response needs, and can inform the effective use of vaccines^2^, antivirals^3^, and other countermeasures (e.g. school closures and social distancing). Despite this, and in contrast to research and development of medical countermeasures, few efforts have been coordinated to improve optimization of modeling and other outbreak data analyses during public health emergencies caused by emerging infectious diseases.

Models played a prominent role in the 2014–2016 Ebola epidemic in West Africa. During the first half of the outbreak, public health operations were complicated by situational awareness limited by sparse data availability from the affected countries^4^. Reports from the ground indicated that the outbreak was growing quickly, but uncertainty about the current and future trajectory of the epidemic slowed mobilization of the public health response^4^. Faced with that uncertainty, decision makers incorporated information from infectious disease models, with early forecasts indicating that incidence would continue to grow rapidly unless aggressive interventions were implemented. For example, a forecast generated by the CDC predicted up to 1.4 million Ebola cases with no additional interventions or changes in community behavior^5^. These forecasts likely contributed to the acceleration of the international response and provided guidance for how resources might be effectively deployed.

Nevertheless, the integration of those analyses into the decision-making cycle for the Ebola 2014–2016 epidemic was not seamless, a pattern repeated across many recent outbreaks, including Zika^6^. Reasons for this vary. Modeling and outbreak data analysis efforts typically occur in silos with limited communication of methods and data between model developers and end users. Modeling “cross talk” across stakeholders within and between countries is also typically limited, often occurring within a landscape of legal and ethical uncertainty. Specifically, the ethics of performing research using surveillance and health data^7^, limited knowledge of what types of questions models can help inform, data sharing restrictions^8^, and the incentive in academia to quickly publish modeling results in peer-reviewed journals contribute to a complex collaborative environment with different and sometimes conflicting stakeholder goals and priorities.

To remedy these challenges, we propose the establishment of ‘outbreak science’ as an inter-disciplinary field to improve the implementation of models and critical data analyses in epidemic response. This new track of outbreak science describes the functional use of models, clinical knowledge, laboratory results, data science, statistics, and other advanced analytical methods to specifically support public health decision making between and during outbreak threats. Outbreak scientists work with decision makers to turn outbreak data into actionable information for decisions about how to anticipate the course of an outbreak, allocate scarce resources, and prioritize and implement public health interventions. Here, we make three specific recommendations to get the most out of modeling efforts during outbreaks and epidemics. Together these recommendations constitute the foundation of an integrative field that is “outbreak science”:

## 1: Be prepared: Establish functional model capability and foster relationships before the emergency begins

In many of the recent communicable disease public health emergencies, a suite of academic and government modelers produced important analyses, but few were specifically tasked and funded to do so in a coordinated, functional fashion. The reactive (rather than prospective) mobilization of modelers and computational epidemiologists in public health emergencies is not new. Rapid development and deployment of infectious disease models was important during the 2009 influenza pandemic, chikungunya, and SARS^9–11^. Models helped decision makers to understand the likely trajectory of the outbreaks, the risk of international spread, and the potential impact of interventions. Although identified as influential at the time, few if any investments in permanent capabilities to integrate such analyses into the decision-making process have followed.

However, a few examples of successful partnerships between modelers and decision makers may serve as inspiration. Small teams exist in agencies in the US government such as at CDC, BARDA and NIH. Similar examples exist in the UK with expertise within government agencies and from academic entities, for instance, Imperial College and the London School of Hygiene and Tropical Medicine.

Leveraging these promising examples (not an exhaustive list), we therefore recommend that epidemic modeling capability be enhanced and honed not during rapidly-evolving public health emergencies, but rather between major epidemics. Such approaches can leverage historical datasets and/or be developed using routine communicable disease data. An excellent example of this is the Epidemic Prediction Initiative (EPI) [https://predict.cdc.gov], which has built a framework for open forecasting and transparent evaluation and facilities participation through standardized data and forecast formats and community building^12^.

## 2: People and computation: Develop connections between model developers and model users

Currently, limited information is available on how those on the frontlines of public health perceive and use models in epidemic decision-making. Understanding the functional relationship between the public health end-users and model developers is critical to improving capacity during outbreaks. A recent pilot study (*n* = 29 respondents) in Australia suggests a disconnect between model end-users and model developers, with overall infrequent use of modeling in public health practice due to factors such as a lack of in-house modeling expertise, variable trust and/or comprehension of model outputs, and uncertainty regarding whether models could answer the most important operational questions on the most relevant temporal and spatial scale^13^. Similarly, state and local health officials in the US reported reluctance in translating pandemic influenza epidemic models into public health action, despite the respondents’ familiarity with such models^14^. More recently, a limited evaluation of 39 influenza public health practitioners from three US public health organizations indicated that approximately one in five had direct communication with modelers, 67% felt that models should be more relevant to public health questions, and 59% wanted more frequent communication with model developers^15^.

But this relationship must also be a two-way street. Without opportunities to gain experience, modelers do not necessarily understand or anticipate the needs and preference of decision makers. Improved communication and collaboration between the two stakeholder groups would improve the quality and utility of models and the decisions they support. This suggestion is not novel, yet the need clearly remains in 2019^13,15^. To improve these connections, we advocate systematically measuring perceptions of both model end-users and model developers to identify what are the key barriers to functional model development and implementation. For example, stakeholder surveys, focus groups and other qualitative data can identify whether the public health priorities as interpreted by model developers are aligned with end-users, whether end-users trust or prefer particular models (or modelers) over others, and whether major gaps are present in end-user modeling literacy and/or model developer communication.

## 3: Modelers on the front-lines: Formalize ‘outbreak science’ by building capacity and supporting training and practice opportunities

Developing and implementing epidemic models under the “fog of war” is an enterprise far removed from the controlled conventional academic setting of epidemiology and biostatistics. We call for cross-cutting outbreak science fellowship programs in which epidemic modelers partner with experts from other disciplines including clinicians, basic scientists, computational biologists, veterinarians, entomologists, epidemiologists, environmental scientists, and those in operational public health. Ideally, these programs would involve rotations in the field to experience challenges like rapidly changing information, varying quality of data, critical inter-personal relationships between and within institutions, and evolving public health priorities. The CDC’s Epidemic Intelligence Service program, a good model for training public health practitioners in applied epidemiology, could serve as a template for a similar program that provides academic modelers and other stakeholders with training in applied infectious disease modeling. Furthermore, training programs of this kind can facilitate research opportunities, as called for in recommendation #2, that investigate relationships and processes between modelers and end users, document current challenges to model implementation during epidemic response, and identify best practices needed to promote integration of modeling into public health.

## Conclusion: Invest in functional modeling capability before the emergency begins

To make outbreak modeling a more effective component of decision-making during outbreaks requires a paradigm shift. To be successful, building working relationships is as necessary as building quantitative modeling capacity. Rather than waiting for the next pandemic, we should use opportunities between pandemics to fine tune model development and implementation and formally measure and redress any disconnect between model developers and model-users.

Finally, we see great potential for a new track of epidemic modeling we have dubbed ‘outbreak science’ to improve public health preparedness response. With sufficient investment in the development and growth of the field, those trained in ‘outbreak science’ will become vital contributors in helping public health professionals navigate the many difficult decisions that come with infectious disease emergencies. Additionally, they will serve as a key link between quantitative modelers and public health decision makers in an era of increasing epidemic threats.
